# Postzygotic Somatic Mutations in the Human Brain Expand the Threshold-Liability Model of Schizophrenia

**DOI:** 10.3389/fpsyt.2020.587162

**Published:** 2020-10-22

**Authors:** Shiva M. Singh, Christina A. Castellani, Kathleen A. Hill

**Affiliations:** Molecular Genetics Unit, Department of Biology, The University of Western Ontario, London, ON, Canada

**Keywords:** neurodevelopment, *de novo* mutations, postzygotic somatic mutations, epimutations, neuronal diversity, mosaicism, neurological disorders, threshold liability model

## Abstract

The search for what causes schizophrenia has been onerous. This research has included extensive assessment of a variety of genetic and environmental factors using ever emerging high-resolution technologies and traditional understanding of the biology of the brain. These efforts have identified a large number of schizophrenia-associated genes, some of which are altered by mutational and epi-mutational mechanisms in a threshold liability model of schizophrenia development. The results, however, have limited predictability and the actual cause of the disease remains unknown. This current state asks for conceptualizing the problem differently in light of novel insights into the nature of mutations, the biology of the brain and the fine precision and resolution of emerging technologies. There is mounting evidence that mutations acquired during postzygotic development are more common than germline mutations. Also, the postzygotic somatic mutations including epimutations (PZMs), which often lead to somatic mosaicism, are relatively common in the mammalian brain in comparison to most other tissues and PZMs are more common in patients with neurodevelopmental mental disorders, including schizophrenia. Further, previously inaccessible, detection of PZMs is becoming feasible with the advent of novel technologies that include single-cell genomics and epigenomics and the use of exquisite experimental designs including use of monozygotic twins discordant for the disease. These developments allow us to propose a working hypothesis and expand the threshold liability model of schizophrenia that already encompasses familial genetic, epigenetic and environmental factors to include somatic *de novo* PZMs. Further, we offer a test for this expanded model using currently available genome sequences and methylome data on monozygotic twins discordant for schizophrenia (MZD) and their parents. The results of this analysis argue that PZMs play a significant role in the development of schizophrenia and explain extensive heterogeneity seen across patients. It also offers the potential to convincingly link PZMs to both nervous system health and disease, an area that has remained challenging to study and relatively under explored.

## Introduction

Mutations provide the foundation for individual genetic differences. They also play a critical role in health and disease as their effect on an individual may range from being lethal to beneficial. Although most genetic variations in populations and families are passed on from generation to generation, some may be acquired *de novo*. Traditionally, *de novo* mutations of interest to diseases have been identified by the genomic difference between parents and the progeny with the disease. The assumption is that the mutation causing the disease in the progeny must have arisen in one of the parental gametes used to generate the zygote and the resulting progeny. Such *de novo* germ line mutations may become part of the familial gene pool. The occurrence of *de novo* mutations however is not restricted to the germ line, only. Most represent errors in DNA replication that may follow meiosis to generate gametes or mitosis to increase the number of somatic cells during development and differentiation of the zygote. Theoretically, postzygotic *de novo* mutations may originate at any time during development, from the early two-cell dividing embryo to any stage of an individual's prenatal and postnatal development. Also, cells carrying *de novo* postzygotic mutations may become part of an individual's heterogeneous genomic composition. As such, they have been difficult, even daunting to identify and appraise. Improved technologies and innovative experimental designs, such as single-cell genome sequencing, have revolutionized this research. For the first time it is possible to identify postzygotic mutations, with increasing sensitivity using ongoing revolutions in genomic technologies (1–7).

Postzygotic somatic mutations may contribute to mosaicism and add another layer of genetic variation across individuals, potentially affecting physiology, function and phenotype. Although it was once assumed that such mutations are rare and inconsequential they may be almost two orders of magnitude more frequent than germline mutations (8). Yet, the degree and consequences of mosaicism resulting from somatic mutations have not been adequately assessed, primarily due to inaccessibility of needed samples and lack of necessary technologies. As such, somatic mutations represent the latest addition to measures of biological diversity, an area of research that until recently relied almost exclusively on germline mutations. Generally, postzygotic development has been characterized by an increase in cell number via mitosis and strictly regulated dynamics in gene expression. The cells produced by mitosis during ontogeny have long-since been considered primarily genomic clones of the zygote. Although mutations representing DNA sequence changes may theoretically arise with every cycle of mitosis (9), the fate of cells carrying such mutations is not assured. If a mutation occurs very early in development, the new mutation may be incorporated in most tissues of the progeny. However, if the mutation occurs later in development, fewer cells will carry the mutation, and the mutation-carrying cells may be confined to a given tissue and/or cell type. Additionally, cells carrying new mutation(s) may be selected against during development, while others may have mutations that result in little to no phenotypic effect (low-level or micro-mosaicism). Alternatively, some such mutations can have a positive effect on cell proliferation and subsequently drive the accumulation of mutant cells. These driver mutations may result in significant mosaicism with potential to affect the phenotype. Additionally, somatic mutations can arise as random events, programmed events, or as the product of an inherited genotype as in the case of a mutator phenotype (10). A long-term consequence of ongoing somatic mutations may be that some/most non-germ cells (soma) will differ in their genomes and thus in theory no two somatic cells may harbor 100% identical genomes (11–13). The consequence is that every individual will embody a composite mosaic of genetically distinct cells. The contributions of somatic mutations and somatic mosaicism were previously not considered biologically significant, and the phenomenon of postzygotic mutation has remained largely underexplored due to the biological complexity of mutagenesis, mosaicism, access of required cells and tissues for evaluation and the technical challenges of single-cell “omics.” Despite these hurdles, the potential of postzygotic mutations has been implicated in a number of diseases [see review, (14)]. Specifically and most relevant to this discussion they have been implicated in a number of mental disorders [see recent review, (15)]. In this overview, rather than offering yet another extensive review on the subject, we offer a measured perspective on the phenomenon of developmental postzygotic mutations relevant to mental disorders and specifically expand upon the threshold model of schizophrenia, a devastating life-changing neurodevelopmental disorder of poorly understood etiology despite the increasing appreciation of the contribution of postzygotic events in complex diseases (reviewed elsewhere).

## Somatic Mutations are Heterogeneous and Accumulate Over Time With Effects on Neurological Phenotypes

Empirically, somatic mutations leading to mosaics with different genotypes within an individual have been recognized over the decades (16), but the nature and extent of the mosaicism is almost never known (2). Rare studies that have begun to characterize the origin, nature, and consequence of somatic mutations and mosaicism show that the rate of *de novo* somatic mutations leading to mosaicism differs with age and across tissues, individuals, and families (2, 4, 17–20). Also, somatic mutations may arise as single-nucleotide substitutions, transpositions, insertions, deletions (including copy number change), and aneuploidies. These events appear at random and are likely caused by a variety of mutational mechanisms. Some of the mutational mechanisms may be development-specific while others may constantly add new mutations throughout life in a clock-like manner (9). The rate and propensity of somatic mutations may also differ across genes and gene sequences (20). Interestingly, despite the assumption that such mutations are stochastic, there is emerging evidence that some somatic mutations may be programmed, as in the case of somatic hypermutation involving immunoglobulin genes (21–25). Developmental windows for sensitivity to mutational events have also been demonstrated in mouse neuronal cells (26). Such results allow us to hypothesize that postzygotic somatic mutations have the potential to contribute to a broad spectrum of neurological phenotypes from health to disease but remain unexplored. To this end, the better understood dominant role of somatic mutations in cancer could be used as a working model that can be applied to the involvement of PZMs in neurological phenotypes and disorders.

## Postzygotic Somatic Mutations Contribute to Disease: The Cancer Model

Initiation and progression of different forms of cancer represent two of the most explored areas of *de novo* genetic changes in somatic cells over the last few decades (27, 28). This extensive research has identified a long list of critical genes that are reported to undergo somatic changes in variety of cancers (http://cancer.sanger.ac.uk). The results offer a comprehensive resource for exploring the impact of gene-specific somatic mutations, both individually and in combination, across different forms of cancers. Follow-up comprehensive studies on such genes have provided the precise role of these mutations in oncogenic transformations. Some of these insights have been possible via increased resolution involving studies on single cells that include single-cell sequencing (29) and single-cell multiomics (30). The results of these studies have assisted in uncovering molecular insights relevant to the initiation and progression of different types of cancers (31–33). Of special interest to this discussion is the role of *de novo* mutations acting as the “second hit” that may initiate and subsequently serve as the “driving force” behind unchecked proliferation and progression of different cancer phenotypes (34), including metastasis (35). Also, *de novo* mutations have been observed to increase at a constant rate with each genome replication in mutator phenotypes (36) or increase sporadically with transient hypermutability (31, 32) in different forms and stages of cancer (37–39). These results have allowed characterization of subtypes of cancers based on signatures of *de novo* mutations and identification of critical players at different stages in carcinogenesis (40). Translationally, such insights have helped to redefine cancer from an untreatable, poorly understood disease to one that can be classified, diagnosed, treated, and in some cases, cured (40), thus adding new hope to what would otherwise be a bleak diagnosis just a few years ago. We note that neurological aberrations are currently poorly understood but may involve postzygotic somatic mutations that can be detected and classified. As in the case of cancer, they may provide a valuable starting point toward gaining insight into the etiology of specific neurological disorders. It may lead to the characterization of gene specific changes including somatic mosaicism that may translate to treatment successes for neurological disorders as exemplified by the cancer model.

## Postzygotic Somatic Mutations Often Arise During Neurodevelopment

**Figure 1:**
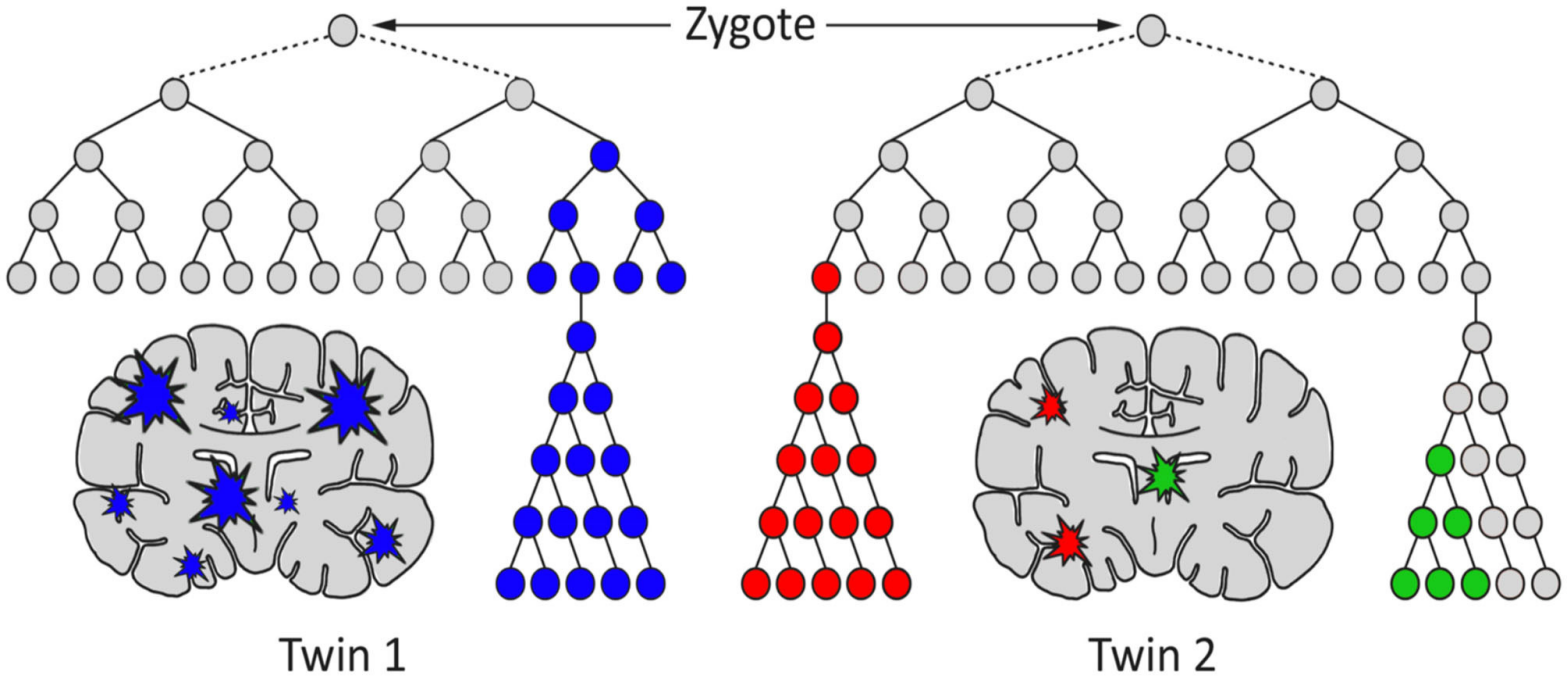
A hypothetical model for the generation of genetic differences between brains of monozygotic twins by postzygotic mutations (PZM). The timing, genes affected, clonality, and brain region(s) affected may cause the twins to develop discordance for neurodevelopmental diseases, including schizophrenia.

**Figure 2:**
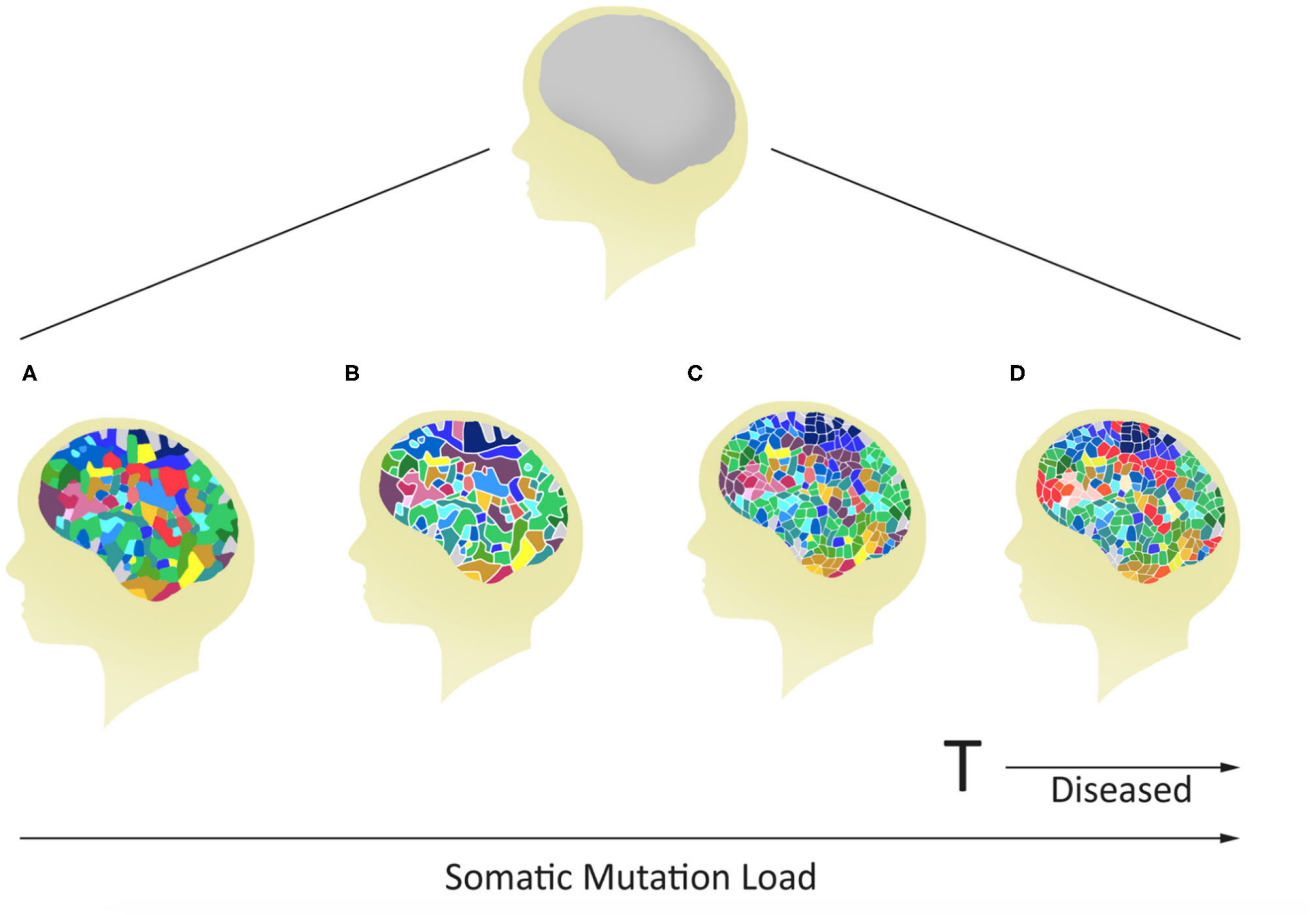
Potential for variable mosaicism in an individual brain based on differences in postzygotic somatic *de novo* mutations during development. The outcome will make every brain unique, some below **(A–C)**, and others above **(D)** a genomic threshold for the development of a mental disease.

Often it has been assumed that all cells in the brain have identical genomes. However, postzygotic somatic mutations arising during the development of the brain have the potential to affect large and small clonal lineages depending upon the developmental timing of the mutation and the resultant cell populations carrying the *de novo* mutation (41). They may generate differences between the neuronal structures of monozygotic twins that started life as a single zygote (Figure 1). Also, they could contribute to the complex spectrum of brain phenotypes across individuals in the population including some disease phenotypes (Figure 2). Heterogeneous PZMs are often reported in the brain (41–43), but it is not known whether they arise through a random process, if they are inherently directed, or if they occur in response to environmental cues. What is understood, however, is that new mutations are somatic, present in the brain and are not directly transmitted to the next generation. PZMs however, may ensure that neuronal genomes in an individual are not singular, homogeneous, or static, but instead establish mosaic, heterogeneous and dynamic populations of neural genomes, with new neurons arising throughout life (42). Among other processes, neurons are known to undergo *de novo* long interspersed nuclear element (LINE-1/L1) retrotransposition (44), as has been reported in adult neurons (45, 46) and during embryonic development (43, 47, 48). Interestingly, the number of retrotranspositions has been reported as significantly higher in brain than non-brain tissue samples (49). Researchers have argued that every cell in the human brain may contain a number of somatic insertions and that retrotransposition may play an important role in reshaping the genetic circuitry (50). This phenomenon can lead to neuron-to-neuron variation, a neuron-specific transcriptome, and a neurobiological phenotype (51–53). Besides retrotranspositions, neuronal variations may also arise from additional genetic alterations in whole chromosome numbers (54), rearrangement of mobile elements (50), insertions/deletions (55), and single nucleotide variants (SNVs) (18). Theoretically, there is no limit to the kind and amount of genomic variation within an individual, and therefore every gene in the human neural genome may be mutated in some neurons (56). The potential exists to uniquely define nearly 100 billion neurons and over 100 trillion neuronal connections. In this context, PZMs will add an extra level of variation for plasticity, adaptability, and resilience to the dynamics of environmental change and insults, which is specifically operational and sensitive in the brain (53). Such results argue that PZMs may be key contributors to intra-individual variability that may affect neuronal phenotypes. In doing so, they also provide a novel and unique insight in the biology of the brain, each representing a unique composite of mosaics that has remained unexplored. What is being realized is that postzygotic transposition events are higher in some mental disorders (49). These events preferentially affect genes associated with neuronal functions, and an uncontrolled retrotransposition may increase the risk of mutations leading to disorders. Indeed, pre-existing retrotransposons may act as “lightning rods” for novel insertions, which may modulate gene expression (49). Specifically, *de novo* PZMs have been reported in a number of neurodegenerative (57, 58) as well as neurodevelopmental diseases (59–61). Of special interest to this discussion is the high frequency of *de novo* mutations reported in patients for neurodevelopmental disorders such as autism (62) and schizophrenia (63–66). Here, we will specifically focus on schizophrenia as a model of neurodevelopmental disorders in assessing the involvement of PZMs in brain disorders.

## Postzygotic Mutations are Candidate Contributors to Schizophrenia

Schizophrenia is a complex neurodevelopmental disorder that is present worldwide at a relatively stable frequency (~1%). The disease is heterogeneous, often expresses in early adulthood and difficult to early diagnose in absence of any biological test. It has high heritability (80%), as well as high discordance in monozygotic twins (50%) (67). The search for genes and inherited factors causing schizophrenia has been long and exhaustive but the identification of causal gene(s) has been elusive. However, this meticulous and long research has identified a large number of schizophrenia-associated single nucleotide polymorphisms (SNPs) and copy number variations (CNVs) in hundreds of genes and non-coding loci (http://www.szdb.org). As it stands, most of the findings reported in this database have been difficult to replicate and are not unique to this disease (68). Some of these genes and mutations could provide predisposition diagnostic information including clinical spectrum for schizophrenia and other related disorders. Further, an assessment of results by the Schizophrenia Working Group of the Psychiatric Genomics Consortium (2014) has identified 108 of the most common gene variants that have met a high degree of significance but explain a small fraction of the disease risk. Yet additional analysis has led to the prioritization of 145 most common schizophrenia variants that may serve as a foundation for patient specific genetic liability for this disease (69). These variants may directly or indirectly contribute to a disease threshold that could be achieved by inherited variants in concert with still-uncharacterized random events, including the effect of the environment. Indeed, most research on the development of schizophrenia is compatible with this long-standing threshold model (70, 71).

Of the random events reported in schizophrenia, two biological features appear noteworthy. The first involves *de novo* DNA sequence changes including CNVs reported in the brain (72) and blood (73–75). These often affect synaptic genes, and associated CNVs tend to occur in genome regions that are prone to recurrent mutations (76) and are implicated in schizophrenia (77, 78). The second random feature implicated in schizophrenia is epimutations, particularly changes in DNA methylation (79). They may undergo such changes randomly or in response to external exposures and be passed on through succeeding mitotic cycles (9, 16). Epimutations, that often affect gene expression may complicate familial tendencies with random and environmentally-responsive events. Additionally, there may be a role for epigenetically-regulated human endogenous retroviral HERV and related sequences (80). HERVs are retroviruses that have the potential to transpose and facilitate copy number changes. They may also affect epigenetic features (80). Such sequences have been uniquely isolated from the genomes of the affected members of the monozygotic twin pairs discordant for schizophrenia (MZD) (81). Additionally, HERV-related DNA/RNA sequences that were detected in the genomes of the affected members of MZD for schizophrenia have been reported to be elevated in schizophrenia patients (82). These sequences may represent copy number changes and/or increased expression with potential to increase transposition. Additionally, the HERV-associated C4 locus within the major histocompatibility complex on chromosome 6 may affect C4 expression, which is involved in the pruning of the dendritic spine in schizophrenia (83). These results support the hypothesis that schizophrenia may involve mutations representing DNA sequence changes as well as changes representing any epigenetic modifications that may affect gene expression. Together, they may function as the sum total of postzygotic somatic modifications (PZMs) that may contribute to the development of schizophrenia. Some of these modifications may be hereditary and transmitted through the parent(s), while others may represent postzygotic changes that are now technologically identifiable (using exceptional methods) and allow for proposition of an expansion of the threshold-liability model for schizophrenia.

## An Expanded-Threshold-Liability Model for Schizophrenia May Include Postzygotic Events

**Figure 3:**
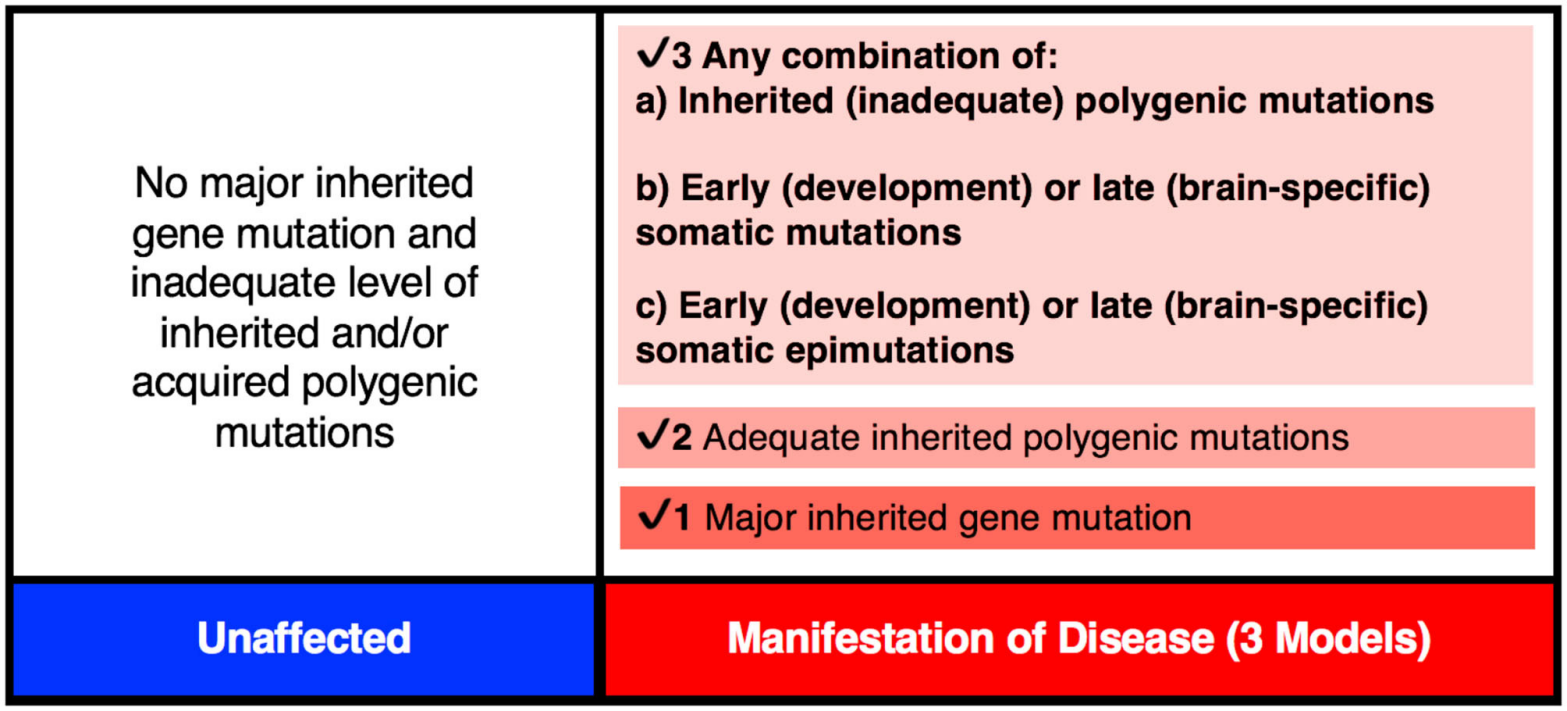
The genetic threshold required for the development of a neurodevelopmental disease may be met by (1) A major gene insult; (2) An adequate level of polygenic mutations; or (3) A combination of inherited (a) plus acquired somatic mutations (b) and/or somatic epimutations (c).

There is mounting evidence that every brain is a unique mosaic representing a composite of genetically distinct cells [see review (83)]. It may apply to a “normal” brain as well as a brain from an individual with a neurological disorder, including schizophrenia (56). Here, the brain from a patient may carry many more mutations/modifications affecting pathway(s) defective in schizophrenia. Also, given extensive heterogeneity, the number and type of genes affected may be unique across all/most patients with schizophrenia. Brains from different patients will be expected to carry heterogeneous sets of mutations/epimutations and these differences may account for the highly variable disease manifestation across patients. In some patients, the set of causal mutations/modifications needed for disease manifestation may all be inherited from parents, while in others they may represent inherited plus somatic *de novo* events that may include DNA sequence changes and/or epimutations (Figure 3). Here, the *de novo* events may or may not be needed to raise the liability and eventually to cross the threshold of liability for the manifestation of the disease (84, 85). Naturally, the acquired *de novo* neuronal mutations and epimutations will not be transmitted to the next generation even though any predisposition for such mutation(s), if present, may follow a familial transmission. The inclusion of such postzygotic events adds a novel and acquired somatic genetic/epigenetic change during the life of the individual to the causation of the disease.

**Figure 4:**
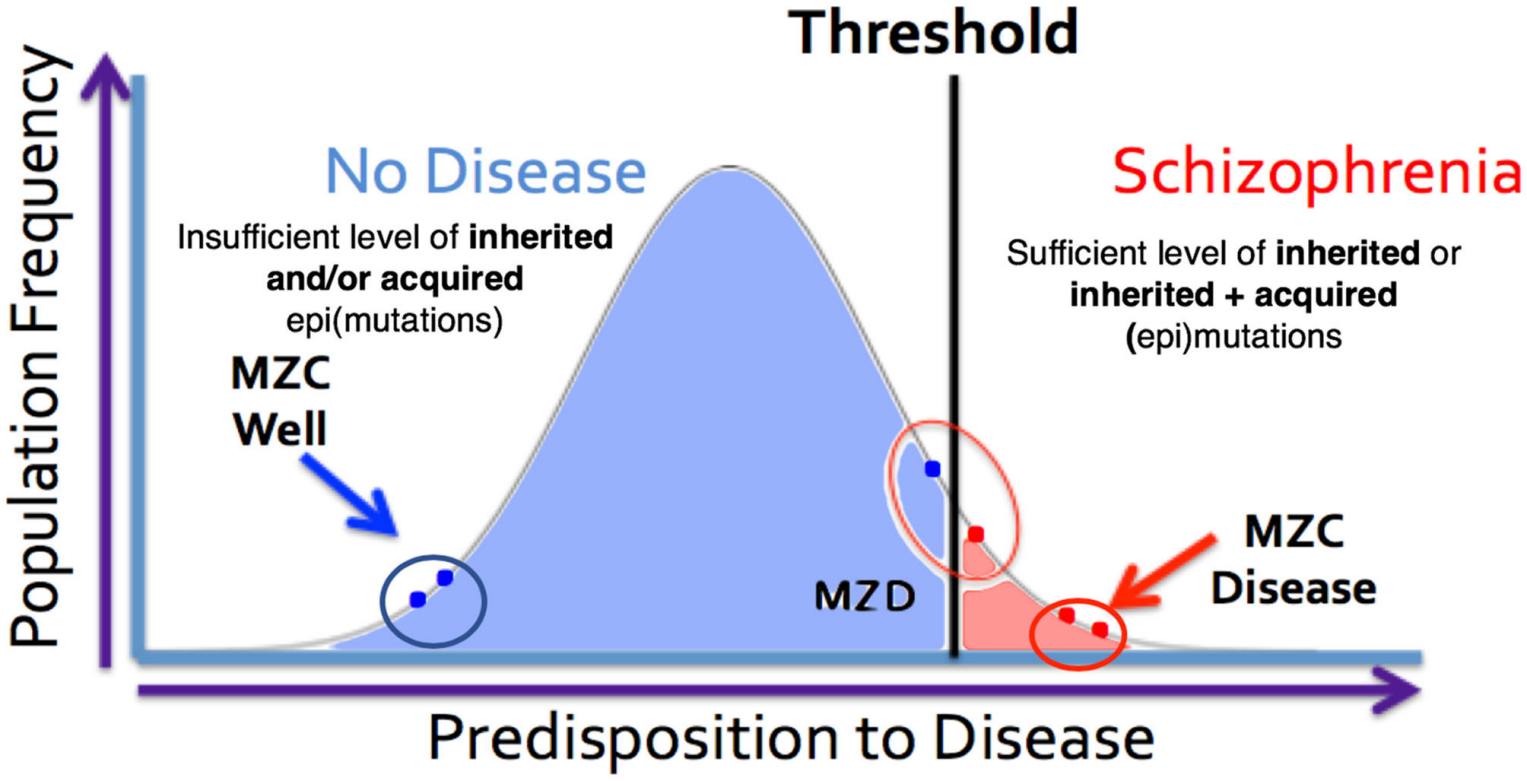
MZ twins as a model for assessing the genetic threshold liability hypothesis. Representation of three sets of twins [MZC well (monozygotic twins both unaffected), MZC disease (monozygotic twins both diseased) and MZD (monozygotic twins discordant)] within the threshold liability hypothesis (unaffected = blue, disease affected = red). In this model, the affected twin in the MZD pair must acquire somatic mutation/epimutation to cross the threshold and develop the disease. Genomic and epigenomic assessment of these exceptional twin pairs will allow for identification of pair-specific postzygotic somatic event(s) [Adapted from Castellani et al. (88, 89)].

The expanded-threshold-liability model for schizophrenia that includes postzygotic mutations and epimutations (PZMs) is compatible with a number of unusual features of this disease. These include high discordance (~50%) of the disease among monozygotic twins, adult onset of the disease, high heterogeneity, and a spectrum of manifestations including overlapping endophenotypes involving different diagnostic entities. For the first time, this model also provides the most logical biological explanation for a comparable risk of transmission of schizophrenia by the members of a monozygotic twin pair discordant for the disease (MZD) (86, 87). The model argues that the affected and unaffected members of the MZD pair would inherit a comparable level of genetic predisposition and pass it on to their respective offspring. This level of inherited liability is expected to be below the disease threshold. However, the addition of *de novo* somatic PZMs (by chance alone) in the disease twin will lead to the development of disease (Figure 4) while maintaining a comparable risk of transmission to the next generation by both members of the MZD twin pair. Here, the risk of transmission of the disease by the ill twin will not be any higher than the risk of transmission by the well (unaffected) twin. Also, this expanded-threshold-liability model being presented for schizophrenia will be applicable to most neurological disorders with high discordance in monozygotic twins. It will be particularly relevant in disorders with neurodegeneration and neurodevelopmental manifestation. Although logical, the expanded model remains a theoretical concept and needs to be tested and established experimentally.

## Testing The Expanded-Threshold-Liability Model for Schizophrenia

Testing the proposed expanded-threshold-liability model in schizophrenia that incorporates *de novo* neuronal postzygotic mutations and epimutations, acquired during the life of an individual will be challenging for two reasons. First, the somatic *de novo* events that add to the threshold for the disease will not be transmitted to the next generation; they are acquired by an individual during his/her lifetime and are eliminated from the population with their death. Second, the somatic nature of *de novo* mutations will generate complex mosaicism, making every brain genetically heterogeneous, different and unique (41). This cellular heterogeneity will present a special challenge in any attempt of characterization as it will require assessment of cell-specific, rather than brain-specific, features. To this end, a variety of evolving methods and experimental designs including single cell genomic and multiomics (61, 90) may offer novel strategies and resolution to this complexity. Indeed, it will be challenging to identify and obtain desirable cells for such studies. Taking just any cell from any individual or brain will not be satisfactory. There will be a need to compare the neuronal zygotic and postzygotic genome that is not always practical or ethically acceptable. Although the use of multiple tissues is a practical way to identify postzygotic DNA sequence changes, others including epigenetic changes and transpositions may present problems as they are expected to be tissue specific. The challenges in ascertaining the extent and nature of acquired liability will also relate to the timing of mutation, the size of mutant clones, the accessibility of the mutant cells, the cell types affected, and the positional localization of the mutant cells. Additionally, it will be challenging to distinguish disease-relevant mutations from background *de novo* variants necessitating interpretation using a tested statistical framework (91). Under these circumstances, there is an imperfect alternative that may help assess the feasibility of the expanded-threshold-liability model in schizophrenia. Here, monozygotic twin pairs discordant for schizophrenia (MZD) may be used to represent an appropriate resource for testing the expanded-threshold-liability model for schizophrenia (88, 89). Recall that members of the MZD pair begin life as a single zygote but develop independently as two different individuals (somas). They share common familial predispositions and acquire independent PZMs, making the two twins distinct (Figure 1). Here, the most logical experiment involving a direct assessment of brain regions potentially carrying different somatic mutations/epimutations in the two brains will be difficult for a variety of reasons including accessibility. However, any PZM that occurs very “early” during embryonic development may be expected to be maintained in neuronal as well as (some) non-neuronal cells during ontogeny. As such, some of the easily accessible non-neuronal cells (blood, cheek swabs, etc.) may serve as a proxy for occurrence of “early” somatic mutations that may also be present in neuronal cells. Indeed, DNA from the blood of MZD twins has been shown to be not 100% identical (74, 89, 92). They carry a variety of sequence differences, epigenetic and genetic, that must result from postzygotic events. Here, any use of blood DNA from MZD twins could overcome challenges faced by the inaccessibility of live neuronal cells for evaluation, even though such results will be limited and may miss out on “late” mutations that may have occurred after the differentiation of a neuronal lineage. Also, the genome of the unaffected twin may be used to represent the zygotic genome that started the two members of the MZD twin toward identification of any/all schizophrenia specific postzygotic changes in the disease twin. This is by no means a perfect experiment. However, the results will have the potential to implicate involvement of somatic mutations in the genes that are known to be involved in the disorder (69), particularly those that occurred “early” during the independent development of the two twins.

Interestingly, the complete DNA sequence and genome-wide DNA methylation (88, 89) data on the blood DNA from two MZ twin pairs discordant for schizophrenia and their parents available in the literature allows us to assess any involvement of PMZs in this disease. The analysis of these data has allowed an assessment of a large number of disease associated mutations (http://www.szdb.org) reported in the blood DNA. Specifically, it has allowed assessment of postzygotic changes in the 145 most common schizophrenia associated gene variants (69) in the disease twin that is not present in his/her unaffected counterpart. The family specific genome sequence results (89) show that every member of the two families carried a subset of common schizophrenia-associated gene variants (69). Although the majority of the schizophrenia-associated gene mutations present in the twins are shared and present in one or the other parent (inherited), the remainder are not seen in either parent and are unique to only one member of the MZD twin pair (89). The latter are most compatible with their postzygotic origin. More important, they could have not originated in any parental gamete that produced the zygote. The results also show that although both members of the MDZ twin pairs carry some disease-related mutations, the affected members have acquired additional disease-associated mutations in glutamate and dopamine pathway genes in both patients [see details in (89)]. It is argued that such mutations will have the potential to help cross the disease liability threshold and develop schizophrenia in the diseased twin only (Figures 3, 4). It is important to suggest that any dataset that is based on blood DNA is limited to known schizophrenia-associated *de novo* events that may have occurred “early” in development. As such, they may not include *de novo* mutations that may have occurred “late” in development, not apparent in the blood and would be/are confined to the brain.

It is important to note that not all mutations that may contribute to schizophrenia involve sequence changes. Some other forms may involve epigenetic changes including DNA methylation. The details of genome-wide methylation results on the same two MZD pairs that were studied for genome sequence difference reported in Castellani et al. also show methylation aberrations in the blood DNA of the patient as compared to the well member of the twin pair (88). The results show that *de novo* events involving genome sequence and epigenetic DNA methylation changes may be independent and add to the disease liability in an expanded-liability-threshold model of schizophrenia. To the best of our understanding such results provide among the most comprehensive account of all/most gene mutations and DNA methylation changes that may have led to the development of schizophrenia in the two affected members of the two unrelated MZD twins. They may represent a near complete list of disease-causing mutation in individual patients for the first time. It has been made possible by assessment of the monozygotic twins discordant for schizophrenia, where the genotype and epigenotype (methylation specificity) of the well-twin is used to serve as the perfect-matched control. It allows a reliable assessment of inherited as well as postzygotic somatic changes during the independent development of the members of the MZD twin pair that started life as a single zygote. Finally, it is critical to note that these results are based on only two patients. There is a need to replicate such results on a larger sample size. We note that it will be challenging to find and study such patients belonging to well-characterized MZD pairs. Indeed access and availability of brain DNA from such MZD twins for such studies will be most valuable. To this end, we recognize the challenge and our inability to acquire a more comprehensive result that includes all genetic and epigenetic variants, inherited, acquired, and present in different regions of the disease brain. Such results however, will be needed to fully define the nature of genetic predisposition in individuals, including the unaffected member of the MZD pair. Despite such concerns, the limited results discussed offer potential involvement of PZMs (89) that includes epimutations (88) in the development of schizophrenia.

It is important to point out that the PZMs implicated in schizophrenia affect a relatively large number of genes, and that not all patients will require postzygotic changes during development in order to reach the disease threshold. In some cases, all of the changes necessary for the manifestation of the disorder will be acquired via familial transmission. This may be the case in monozygotic twin pairs that are concordant (MZC-disease) for the disease (Figure 4). The proposed expanded model is testable using MZD twin pairs that have inherited some, but not sufficient, genetic liability to reach the disease threshold. Unlike the unaffected member of this MZD pair, the affected MZD patient is shown to have acquired additional PZMs leading to a threshold necessary for disease manifestation (Figures 3, 4). The addition of *de novo* somatic mutations and epimutations during early or late development (and present in the brain) is a timely addition to the revised threshold-liability model for schizophrenia. This expansion recognizes that almost all neurodevelopmental disorders are multifactorial and have a heterogeneous causation. These may include any of the three options: major familial mutation(s), adequate inherited polygenic mutations/epimutations, or a combination of inherited and acquired *de novo* (early and/or late) mutations and epimutations that help cross the liability threshold for the development of schizophrenia. This expanded model should be applicable to most disorders with complex genetic and epigenetic etiology and involvement of postzygotic changes.

One of the major challenges in neurologic disease research is the accessibility of appropriate target biological sample and use of perfectly matched control. Such samples are particularly critical for characterizing neurological disorders, including schizophrenia. These disorders involve a large number of genes, where each gene itself contributes relatively small effects. Most of the genes involved are expected to be polymorphic in the population, and many of the genes may undergo postzygotic genetic and/or epigenetic changes, particularly in the brain. This extensive polygenicity and inherited and non-inherited heterogeneity makes the investigation of the etiology of neurological disorders one of the next great challenges in biomedical science. Of special concern is the complex genetic and epigenetic somatic mosaicism being increasingly reported in the brain. Although logical and timely, research on *de novo* mutations and epimutations will demand novel approaches and high-resolution technologies, clever experiments, exceptional patients, precious and ethically sensitive biological samples, ample time and resources. Additionally, many of the specific PZMs reported to date have not yet been replicated, cataloged and curated. Here, the proposed Brain Somatic Mosaicism Network (93) has the potential to open novel avenues that have remained unexplored. This initiative will include refinement of technologies (94) that will permit characterization of every neuronal type (95) and its connectivity with other neurons in the mammalian brain (96). The anticipated results will be revolutionary and provide a long-awaited breakthrough for the field of precision medicine in neurodevelopmental disorders, and in particular, schizophrenia. Among the most immediate application of precision corrections may include *in vivo* neuroepigenome editing for treating brain pathology (97).

## Concluding Remarks

The mammalian brain is a dynamic organ with a high degree of mosaicism likely caused by postzygotic genetic and epigenetic alterations that may contribute to most multifactorial and complex neurological disorders. Traditionally, researchers have used an established threshold-liability model that incorporated the sum total of all inherited mutations in combination with environmental factors to define a liability scale sufficient for the development of disease (Figure 4). As it stands, this model was postulated before there was any realization of any role for postzygotic genomic/epigenomic changes and mosaicism. The timely revision of this model included in this discussion incorporates two observations. First, postzygotic changes are rather widespread and make every brain a unique genetic mosaic (Figures 1, 2). Second, depending on the number and type of genes affected, the postzygotic changes may contribute to the liability scale toward the threshold of the development of a disorder like schizophrenia (Figure 3). The occurrence of differential postzygotic changes (both DNA methylation and DNA sequence) has been demonstrated in the MZD twin blood DNA (88, 89). It may serve as a proxy for their differential presence in the brain. The observed PZMs seen in the blood of MZD pairs are expected to have occurred “early” during development and differentiation but this must be shown empirically.

The results discussed here are compatible with the involvement of postzygotic somatic changes in schizophrenia. A comprehensive assessment of this phenomenon will only be possible if all early, late, and ongoing postzygotic alterations present in the brain are identified. Further studies will require the assessment of postmortem or surgery-derived samples of brain regions relevant to the disorder. Some of these may become feasible given the need for relatively small number of cells needed for this assessment as future experimental work must use more refined methods including single-cell multiomics at the neuronal level. An informative experiment may involve single-cell neurons from monozygotic twins discordant for the disease. Also, there may be a need to add the genomic location, cells representing relevant brain region(s), and the genetic background of the individual to this the “expanded-threshold-liability model.” Finally, the revised model explains the extensive variability in brain phenotypes in the general population and the high discordance of monozygotic twins (MZD) including the fact that both members of the MZD pair have comparable risk of passing on the predisposition to their progeny. Experimental validation, although logical, timely and promising, continues to be challenging. The results, however, will bring about a long-awaited revolution in the understanding of neurological disorders, and their management, prevention and treatment that may include *in vivo* neuroepigenome editing (97). There is every reason to envision that the outcomes of the expanded research direction on schizophrenia will be comparable and even surpass the revolution of precision medicine being realized in the treatment of different forms of cancers. There are major challenges with neurological disorders, but breakthroughs are possible with the right ideas.

## Author Contributions

SS and KH wrote the first draft of the manuscript. CC, SS, and KH contributed equally to the formatting, editing, revision, figures, and intellectual content of the manuscript. All authors contributed to the article and approved the submitted version.

## Conflict of Interest

The authors declare that the research was conducted in the absence of any commercial or financial relationships that could be construed as a potential conflict of interest.
